# Incidence of Heterotopic Ossification after Surface and Conventional Total Hip Arthroplasty: A Comparative Study Using Anterolateral Approach and Indomethacin Prophylaxis

**DOI:** 10.1155/2013/293528

**Published:** 2013-06-24

**Authors:** Dario Regis, Andrea Sandri, Elena Sambugaro

**Affiliations:** Department of Orthopaedic Surgery, Azienda Ospedaliera Universitaria Integrata, Piazzale A Stefani 1, 37126 Verona, Italy

## Abstract

The incidence and severity of heterotopic ossification (HO) in two homogeneous groups of patients that received surface replacement arthroplasty (SRA) and conventional total hip arthroplasty (THA) were evaluated retrospectively. Thirty-nine patients undergoing 42 hip resurfacing procedures and 41 primary cementless THAs through an anterolateral approach received a 10-day course of 150 mg/die of indomethacin postoperatively. The median surgical time was 190 minutes and 156 minutes, respectively (*P* < 0.003). At a minimum 1-year followup, the development of HO was assessed on standard X-ray using Brooker grading. Ectopic bone formation was detected in five cases (11.9%, two Brooker grade I and three grade II) in the SRA group and in 14 hips (34.1%, 12 grade I and two grade II) treated with conventional THA, but the difference was not significant (*P* < 0.11). No clinically relevant periprosthetic ossification (Brooker III or IV) occurred in both groups. Although the difference was not statistically significant, the incidence of HO after SRA was lower than conventional THA. More extensive soft tissue trauma, bone debris, and longer operative time in hip resurfacing are not likely to be absolute risk factors for HO. Further investigations including larger patient populations are needed to confirm these findings.

## 1. Introduction

Heterotopic ossification (HO) consists of an abnormal bone formation in soft tissue, which is typically observed following total hip arthroplasty (THA). 

The overall incidence of periprosthetic ossifications has been reported ranging from 5% to 90% [1], though the rate of clinically relevant HO (Brooker grades III and IV) that could be associated with impaired range of motion (ROM) and decreased functional outcome is 9% [2].

The pathophysiology of HO is multifactorial, and well-known predisposing factors in THA are male gender, hypertrophic osteoarthritis, ankylosing spondylitis, and diffuse idiopathic skeletal hyperostosis. However, the amount of soft tissue trauma, depending on muscle retraction and surgical dissection, and operative time are likely to be critical factors [3]. Consequently, due to a wider exposure and bone debris from femoral head reaming, higher rates of overall (range, 26% to 58.3%) and severe (range, 4% to 7.6%) HO after surface replacement arthroplasty (SRA) have been reported [1, 4, 5]. At a minimum 1-year followup, Rama et al. [6] found an increased incidence and severity of ectopic bone formation in SRA compared to conventional THA, hypothesizing the need to routinely adopt preventive measures after hip resurfacing procedures. Similarly, in a recent meta-analysis study, a significantly higher presence of HO was detected in resurfaced hips [7].

Prophylactic administration of nonsteroidal anti-inflammatory drugs (NSAIDs) was demonstrated to be effective in preventing the development of postoperative HO. In a Cochrane meta-analysis performed by Fransen and Neal in 2004 [8], NSAIDs demonstrated the ability to provide a 59% reduction of ectopic bone formation, although gastrointestinal complications have to be considered. Numerous studies have documented the beneficial effects of indomethacin using different dosages and application periods since 1982 after primary THA in mixed patient populations [9]. McMahon et al. [10] and Amstutz et al. [11] showed that a short-course (ten days) of indomethacin prevents the more significant grades of HO and is effective in reducing the incidence of HO after THA in primary cementless and high-risk populations, respectively.

The aim of this retrospective study was to compare the prevalence and severity of heterotopic ossification in two homogeneous series of patients who underwent surface or conventional total hip arthroplasty through an anterolateral approach and received routine prophylaxis using a 10-day course of indomethacin postoperatively.

## 2. Materials and Methods

The investigation has been approved by the local ethical committee on June 8, 2011, and performed in accordance with the World Medical Declaration Association of Helsinki as revised in 2000. Forty-two consecutive hips (39 patients) that were treated with metal-on-metal resurfacing arthroplasties from August 2004 to June 2009 were retrospectively reviewed. There were 28 males and 14 females, with age ranging from 27 to 72 years (median, 60 years). The etiology was primary osteoarthritis (OA) in 28 patients, avascular necrosis (AVN) of the femoral head in seven patients, posttraumatic arthritis and developmental dysplasia in three patients each, and slipped capital femoral epiphysis in one patient. A hybrid metal-on-metal Conserve Plus prosthesis (Wright Medical Technology Inc., Arlington, TN, USA) was used as resurfacing system, with no local prevention procedures to collect bone debris during femoral head preparation. This series involved the initial experience of the surgical team with SRA.

The control group, including 21 males and 20 females (39 patients, two having bilateral involvement), received conventional cementless THA with ceramic-on-ceramic bearings during the same time period. The median age of the population at operation was 67 years (range, 30 to 77 years). The initial diagnosis was primary OA in 24 hips, AVN in eight hips, rheumatoid arthritis in four hips, developmental dysplasia in three hips, and ankylosing spondylitis and posttraumatic arthritis both in one hip. All conventional THAs were performed using the Procotyl L press-fit acetabular cup and the AnCA-Fit femoral stem with modular neck (Wright Medical Technology Inc., Arlington, TN, USA). A ceramic head was used in conjunction with ceramic liners.

Candidates for hip resurfacing were selected on the basis of age and activity level. Therefore, they were predominantly males (66.7%), with a median of seven years younger than patients undergoing THA (60 years versus 67 years, resp.; *P* < 0.002). However, the gender difference between two groups was not significant (*P* < 0.23). No patient in both groups had undergone previous hip surgery. All procedures were carried out with the patient in supine position through an anterolateral Watson-Jones approach. The median operative time was 190 minutes in the SRA series and 156 minutes in the conventional THA series (*P* < 0.003).

The administration of indomethacin was started on the first postoperative day and continued at the dose of 50 mg three times daily for ten days following surgery. All the hips were available for retrospective evaluation at an average of 34 months (range, 12 to 70 months) postoperatively.

Conventional anteroposterior X-rays of the hip with a standard magnification of 115% were taken at a minimum 1-year followup to allow a complete maturation of the heterotopic bone and compared with the immediate postoperative checks. All radiographs were evaluated by a single observer (E. Sambugaro) who was not involved in the surgical procedure, to avoid interobserver variation and outcome bias. The appearance of HO was assessed using Brooker grading [12], which is a common rating scale to score the extent of ectopic bone formation around the hip joint. The Brooker classification system includes a scale from I to IV to estimate the severity of periprosthetic ossification, and it is the most widely accepted method as it provides a fair intraobserver reliability (*κ* = 0.74) [13].

*Statistical Analysis*. The median rate of HO was obtained for both the patient populations and was compared with use of the nonparametric Wilcoxon signed-rank test as data where not normally distributed according to skewness-kurtosis test. Values for *P* < 0.05 were regarded as significant.

## 3. Results

No patients discontinued indomethacin administration before the recommended ten days because of side effects. The mean duration of followup was 34 months (range, 12 to 70 months).

The incidence of ectopic bone formation was 11.9% (five of 42) in the SRA group, and all cases were Brooker grades I (two, 4.8%) and II (three, 7.1%). The overall percentage of heterotopic ossification in the THA group was 34.1% (14 of 41 hips), including 12 (29.3%) grade I and two (4.8%) grade II.

Consequently, hip resurfacing procedures showed a lower ectopic bone formation compared to conventional THAs, though the difference was not statistically significant (*P* < 0.11).

No clinically relevant periprosthetic ossification (Brooker III or IV) occurred in both groups.

Periprosthetic bone formation was not responsible for pain or functional impairment in any of the patients, and no reoperation was required for poor clinical outcome related to the presence of HO.

## 4. Discussion 

Heterotopic ossification is a frequent, potentially severe complication after hip surgery.

In a systematic meta-analysis, the total rate of HO following primary replacement of the hip was seen to be between 5% and 90% in the untreated, mixed patient populations [1]. However, the incidence of clinically significant HO (Brooker grades III and IV) ranges around 9% [2].

Bony formation in the soft tissue surrounding THAs is usually asymptomatic, though it may involve clinical impairment, as limited range of hip motion and pain, leading to unsatisfactory outcome after replacement [14]. Whether the development of HO is a multifactorial process, including several patient-related risks, numerous papers reported that the posterior approach was associated with a lower rate of periprosthetic ossification than the anterolateral or transtrochanteric approaches [15–17]. Moreover, the amount of soft tissue trauma is recognized as a critical risk factor favoring HO occurrence [6].

Surface arthroplasty typically requires a wider surgical dissection compared to conventional THA to expose the acetabular cavity and the proximal femur. Besides the extensive soft tissue damage, bony debris derived during reaming of the femoral head is considered to be an additional risk factor to increased rate of HO. A recent systematic review including 46 studies compared the clinical and radiographic outcomes of hip resurfacing and standard hip arthroplasty, showing a statistically significant higher presence of HO in SRA cases [7]. The incidence of periprosthetic bone formation ranges between 26% and 58.3%, with severe HO in 4% to 7.6% of patients [1, 4, 5].

The increased rates during resurfacing procedures have been associated with the extent of mechanical trauma at the time of operation [18]. The relevance of the surgical technique has been supported by Shields et al. [19] who found that removal of bony debris during femoral preparation reduced the formation of HO from 58.3% to 32.8% by simply using a plastic drape.

Back et al. [1] prospectively reviewed a consecutive series of 220 hip resurfacings at a minimum of two years, assessing an overall HO incidence of 58.63%, and a clinically significant rate of 8.18% (Brooker grade III). Three men required excision of heterotopic bone, two for pain and stiffness, and one for decreased ROM. However, they failed to show significant difference in the functional outcome between hips with and without periprosthetic ossification at the latest followup.

There are only a few studies comparing the prevalence of heterotopic ossification between SRA and THA. Amstutz et al. [20] reported on an earlier generation resurfacing implant (*n* = 135) compared with conventional arthroplasty (*n* = 150) performed using a posterolateral approach in a consecutive series of patients with primary osteoarthritis over a 6-year period, reporting severe HO incidence of 16.3% versus 8.7%. In a randomized controlled clinical trial, Rama et al. [6] demonstrated a significantly higher rate of severe HO (Brooker grades III and IV) in the SRA cohort (12.6%; 13 of 103 hips) than that in the THA group (2.1%; two of 97 hips), grade IV being observed exclusively after resurfacing. All procedures were carried out using a posterior approach. Hence, they concluded to consider routine prophylaxis against HO following surface arthroplasty. Conversely, a retrospective study conducted by Ritter and Galley [21] on 45 patients with bilateral disease who simultaneously have received SRA on one side and conventional cemented THA on the other through a lateral transtrochanteric access found no statistical difference between either types of hip arthroplasty in HO formation overall or in the development of more severe grades. Finally, Nunley et al. [22], using a posterolateral approach, assessed an overall incidence of HO higher in the combined SRA (*n* = 197) groups (6.1%) compared with the THA (*n* = 189) group (2.6%), and severe HO occurred exclusively in the patients undergoing hip resurfacing (2.6%).

Nonsteroidal anti-inflammatory drugs such as indomethacin, ibuprofen, naproxen, diclofenac, and selective cyclo-oxygenase inhibitors have been administered for chemoprophylaxis of HO after THA [3]. The use of NSAIDs to prevent bone formation following hip replacement has been the subject of a Cochrane Review [8]. A total of 16 randomized trials including 4763 patients were evaluated. Postoperative NSAIDs were seen to reduce the incidence of HO between one-half and two-thirds.

Indomethacin is the best investigated and most commonly used drug, and the prophylactic effect against heterotopic ossification after THA has been proven in many studies [23], though a recent review article by Board et al. [24] has concluded that there is currently little evidence to support its routine administration. The major concern with the routine use of indomethacin is related to its potential adverse effects, especially gastrointestinal disorders. However, the decrease in the duration of therapy from the usual 6-week course drastically reduced the incidence of complications, and no patients in our series were withdrawn secondary to side effects. Moreover, the 10-day course of indomethacin was effective to prevent severe ectopic bone formation in both treatment groups.

Undoubtedly, the incidence of heterotopic ossification in THA patients receiving prophylaxis regimen was unexpectedly high, but this could be also related with the use of a ceramic-on-ceramic coupling, as hypothesized by Higo et al. [25]. However, further investigations are required to definitively clarify whether the bearing surfaces may differently affect the rate of HO.

This study has certain limitations. It is retrospective and observational review, involving a low amount of cases. Moreover, the patients were not randomized, as the choice of the procedure was based on age and level of activity. However, the gender difference between the groups was not significant. Nevertheless, the strength of the study includes the occurrence that all patients were operated on through an anterolateral approach, which is known to increase the risk of HO [15–17]. Furthermore, apart from pharmacological treatment, no other prevention procedures (covering drapes and pulse lavage) were carried out to minimize this complication. Finally, to our knowledge a comparative study between resurfacing and conventional hip arthroplasty using anterolateral approach and indomethacin prophylaxis has been never reported previously.

## 5. Conclusions

The present investigation shows that the incidence of ectopic bone formation following surface arthroplasty is lower than conventional hip replacement, both performed via an anterolateral approach and administering short course of indomethacin prophylaxis postoperatively, though the difference was not statistically significant. Consequently, more extensive surgical exposure, bone debris, and longer operative time in hip resurfacing are not likely to be absolute risk factors for heterotopic ossification. Further studies including larger patient populations are needed to definitively confirm these findings.
